# Norovirus Infection and Disease in an Ecuadorian Birth Cohort: Association of Certain Norovirus Genotypes With Host FUT2 Secretor Status

**DOI:** 10.1093/infdis/jiu672

**Published:** 2014-12-10

**Authors:** Ben A. Lopman, Tarak Trivedi, Yosselin Vicuña, Veronica Costantini, Nikail Collins, Nicole Gregoricus, Umesh Parashar, Carlos Sandoval, Nely Broncano, Maritza Vaca, Martha E. Chico, Jan Vinjé, Philip J. Cooper

**Affiliations:** 1 Division of Viral Diseases, Centers for Disease Control and Prevention; 2 Department of Pediatrics, Emory University, Atlanta, Georgia; 3 Laboratorio de Investigaciones FEPIS, Quinindé; 4 Centro de Investigaciones en Enfermedades Infecciosas, Pontificia Universidad Católica del Ecuador, Quito, Ecuador; 5 Institute of Infection and Immunity, St George's University of London, United Kingdom

**Keywords:** norovirus, immunity, histo-blood group antigen, cohort study, incidence

## Abstract

***Background.*** Although norovirus is the most common cause of gastroenteritis, there are few data on the community incidence of infection/disease or the patterns of acquired immunity or innate resistance to norovirus.

***Methods.*** We followed a community-based birth cohort of 194 children in Ecuador with the aim to estimate (1) the incidence of norovirus gastroenteritis from birth to age 3 years, (2) the protective effect of norovirus infection against subsequent infection/disease, and (3) the association of infection and disease with FUT2 secretor status.

***Results.*** Over the 3-year period, we detected a mean of 2.26 diarrheal episodes per child (range, 0–12 episodes). Norovirus was detected in 260 samples (18%) but was not found more frequently in diarrheal samples (79 of 438 [18%]), compared with diarrhea-free samples (181 of 1016 [18%]; *P* = .919). A total of 66% of children had at least 1 norovirus infection during the first 3 years of life, and 40% of children had 2 infections. Previous norovirus infections were not associated with the risk of subsequent infection. All genogroup II, genotype 4 (GII.4) infections were among secretor-positive children (*P* < .001), but higher rates of non-GII.4 infections were found in secretor-negative children (relative risk, 0.56; *P* = .029).

***Conclusions.*** GII.4 infections were uniquely detected in secretor-positive children, while non-GII.4 infections were more often found in secretor-negative children.

Norovirus is increasingly recognized as the most common etiology of diarrheal disease in all age groups and the second most common cause of severe disease in young children. Norovirus is associated with approximately 18% of gastroenteritis cases worldwide, and this proportion is similar across the age range [1]. In some developed countries, including the United States, where infant vaccination has reduced the incidence of rotavirus gastroenteritis, norovirus remains the most common cause of pediatric gastroenteritis in patients brought to medical care [2].

Noroviruses are a genetically diverse group of single-stranded RNA viruses and can be divided into genogroups, of which viruses from genogroup I (GI), genogroup II (GII), and genogroup IV (GIV) infect humans. Immunity is not lifelong, as infection, reinfection, and disease occur throughout life. Challenge studies conducted since the 1970s have indicated that protection against infection and disease is primarily against the same genotype [3]. A recent birth cohort study of Peruvian children confirmed this, as GII infections were protective against subsequent GII infection and disease and reinfection with the same genotype was uncommon [4].

In addition to acquired immunity, there is also a human genetic component to norovirus susceptibility. Noroviruses use glycans of the ABH and Lewis histo-blood group antigen (HBGA) family for attachment to their target cells [5]. Expression of HBGAs is catalyzed by α1,2 fucosyltransferase encoded by the gene encoding α(1,2) fucosyltransferase (FUT2). Individuals with ≥1 functional *FUT2* allele and, thus expression of FUT2 enzyme are called secretor-positive individuals. Homozygous individuals with a nonsense mutation (FUT2^−/−^) are called secretor-negative individuals and represent up to 20% of the European population [6]. Most data on susceptibility to norovirus infection pertain to the prototype Norwalk strain (GI, genotype 1 [GI.1]) used in challenge studies, with limited observational data demonstrating that expression of HBGAs is associated with strain-specific susceptibility to norovirus infection [7–10]. Although most data from field studies suggest a pattern of secretor-dependent susceptibility to norovirus infection [7, 8, 11–13], other studies have shown that secretor-negative individuals can also be infected [14–19]. Several studies have also demonstrated that norovirus strains differ in their ability to bind HBGAs [20–23]. For example, most GII, genotype 4 (GII.4) viruses bind to secretor-positive individuals from all ABO blood groups [14]; GI.1 strongly binds secretor-positive individuals from blood group A, O, and AB [8]; whereas GI, genotype 8 (GI.8) binds saliva from secretor-positive and secretor-negative individuals [23].

While norovirus is frequently detected in stool samples from patients with diarrhea, the virus can also be detected in healthy individuals. Overall, detection rates are approximately 8% among healthy controls [1], but in some studies, mainly involving children from low-income countries, norovirus has been nearly as prevalent, and sometimes more so, in controls than in cases [24, 25]. Cohort studies may be better suited to understand the natural history of norovirus infection and shedding in healthy controls, especially in children in developing countries [24].

In light of the progress of norovirus vaccine development [26], it is becoming critically important to characterize the incidence of endemic, community-acquired norovirus and the acquisition of natural immunity against norovirus infection and diarrhea. In this study, we followed a community-based birth cohort in a rural district in Ecuador with the aim to estimate (1) the incidence of norovirus gastroenteritis from birth to 3 years of age, (2) the protective effect of norovirus infection against subsequent infection and disease, and (3) the association of infection and disease with FUT2 secretor status.

## METHODS

### Ethics Statement

The study was approved by institutional review boards of Pontificia Universidad Catolica del Ecuador (Quito, Ecuador) and the Centers for Disease Control and Prevention (Atlanta, Georgia). Written informed consent was obtained from each child's parent or guardian.

### Study Design, Recruitment, and Data Collection

The study was conducted in Quinindé, a town in Esmeraldas Province in tropical, coastal Ecuador. A diarrhea surveillance cohort of 194 children was recruited between March and December 2009 from within a birth cohort, the ECUAVIDA cohort, which is described in detail elsewhere [27]. Briefly, the cohort of 2404 children from the District of Quinindé was recruited around the time of birth at the public hospital (Hospital Padre Alberto Buffoni [HPAB]) in Quinindé between November 2006 and December 2009. A subset of the larger cohort was included in a group monitored under diarrhea surveillance: children entering the birth cohort between March 2009 and December 2009 who lived within the municipal boundaries of Quinindé were enrolled for the surveillance study and were followed up to 3 years of age. Inclusion criteria into the surveillance study were as follows: (1) healthy and aged <14 days, (2) maternal stool sample availability, (3) maternal age of ≥17 years, and (4) residence in Quinindé. Routine telephone calls were made twice weekly to mothers to determine whether the child in the surveillance sample had diarrhea, and medical records of visits to the cohort pediatric clinic at HPAB were collected. All families were advised on the importance of surveillance and were asked to report all cases of diarrhea to the clinic or directly to the study coordinator in Quinindé. During each telephone call, the mother or caregiver was also asked about any illness at present; respiratory symptoms, fever, diarrhea, or other signs or symptoms in the child or other members of the household were recorded. If diarrhea was reported, the family was asked to take the child to the clinic the same day for assessment of severity, treatment, and collection of a stool specimen. If this was not possible, a physician visited the child's house for evaluation and sample collection either the same day or the next day. Stool collection from asymptomatic children occurred during routine home visits at ages 7, 13, 24, and 36 months and during routine clinic visits at ages 3, 18, and 30 months. A child was considered asymptomatic if they did not have diarrhea for at least 2 weeks before the routine home visit. During home visits at ages 7, 13, 24, and 36 months, child length/height (measured using infantometers/stadiometers) and weight (measured using digital pediatric scales) were recorded.

### Norovirus Testing

All collected stool samples were aliquoted into 2-mL tubes at the FEPIS laboratory in Quinindé and stored at 4°C. RNA extraction was done using the Qiagen QIAamp Viral RNA Mini Kit, and extracted RNA was stored at −20°C prior to analysis. Real-time reverse transcription polymerase chain reaction (RT-qPCR) was used to detect GI and GII norovirus RNA [28]. All cycle threshold (Ct) values of <40 were considered positive; we also repeated all analysis with Ct cutoffs of <35 for GI viruses or <37 for GII viruses. The latter approach reduced the overall rates of infection but did not affect any of the patterns or observed associations (data not shown). Positive samples were genotyped by sequence analysis [29].

### Definitions

A diarrhea sample was defined as a stool sample obtained from a child who had ≥3 liquid or semiliquid stools in 24 hours, with a time of collection of <14 days before the telephone call or consultation with the clinic/hospital. When a child presented to the clinic with diarrhea, stool samples and clinical data were collected after physician evaluation. Norovirus diarrhea was considered an episode of diarrhea in which norovirus RNA was detected in stool by RT-qPCR. Asymptomatic norovirus infection was defined as a stool sample from an asymptomatic child in which norovirus was detected.

### Blood Group and Secretor Genotyping

ABO blood group determination of capillary blood samples was done by agglutination with standard antisera. To ascertain secretor status, saliva samples were collected from children >3 years of age into Oragene Discover OG575 kits (DNA Genotek, Canada), and DNA was extracted by following the manufacturer's instructions. Secretor status genotyping was determined by PCR amplification and pyrosequencing of *FUT2* at nucleotide 428 (G>A) as previously described [6].

### Statistical Analysis

The prevalence and 95% confidence intervals (CIs) of norovirus infection among diarrhea cases and asymptomatic control samples was estimated according to a binomial distribution. We calculated the cumulative incidence of diarrhea, norovirus infection, and disease by Kaplan–Meier survival analysis. Cumulative incidence estimates were generated at 12, 24, and 36 month of age.

The follow-up time for an individual child was divided according to the number of previous infections. For example, the period between birth and first infection was identified as 0 previous infections and the period between first infection and second infection was identified as 1 previous infection. The number of previous infections was then modeled as a time-varying covariate. To examine the association of previous infection episodes, individual-level, and family-level characteristics with norovirus infection and disease risk, we fitted multilevel mixed-effect Poisson regression models. The individual child (with multiple observation periods) was considered the level for the random effect under the assumption of independent covariance. We calculated *P* values for associations with norovirus infection and disease rates and genogroup-specific infection rates by the Wald test. All regression analyses were controlled for age (in 6-month intervals up to 1 year and in yearly intervals thereafter) and sex.

## RESULTS

### Recruitment and Characteristics of Surveillance Cohort

A total of 194 children were recruited and followed up in the surveillance cohort. The 194 children were followed for 206 658 person-days. The range of follow-up was 211–1305 days, with >90% of children followed for at least 2 years. The numbers of children followed to at least 6 months, 1 year, 2 years, and 3 years of age were 194 (100%), 193 (>99%), 183 (94%), and 135 (70%), respectively. A total of 151 children (77%) were recruited on the first day of life, and all were recruited before day 14 of age. Relevant characteristics of study children, their mothers, and their living environment are provided in Table 1. All children lived in an urban environment in the town of Quinindé and tended to be of higher socioeconomic level than the cohort in general (50% were defined as being in the high socioeconomic tertile). A high proportion of children lived in a household with at least 2 other children of households (88 [45%]) and had a flushing toilet (122 [63%]), but relatively few had access to potable piped water (25 [13%]). Blood group and secretor genotypes were determined at 3 years of age in 178 children (92%; Table 1): most children (111 [62%]) were blood group O, and 158 (88%) were secretor positive. The proportions, by *FUT2* genotype, were as follows: SeSe (homozygote secretor-positive children), 79 (44%); Sese^428^ (heterozygote secretor-negative children): 79 (44%), and se^428^se^428^ (homozygote secretor negative children): 21 (12%).

**Table 1.** Characteristics of Surveillance Birth Cohort (N = 194)

Characteristic	Individuals, Percentage (No.)
Mother	
** **Age, y, median	24
** **Education level	
** **Incomplete primary	11 (21)
** **Complete primary	59 (114)
** **Complete secondary	30 (59)
Household	
** **Socioeconomic status	
** **Low	17 (33)
** **Medium	33 (65)
** **High	50 (96)
** **No. of other children	
** **0	29 (56)
** **1	26 (50)
** **≥2	45 (88)
Child	
** **Sex	
** **Female	54 (105)
** **Male	46 (89)
** **Birth weight, g	
** **<3300	47 (92)
** **≥3300	53 (102)
** **Breast-feeding duration, mo	
** **0–6	16 (31)
** **7–12	39 (75)
** **≥13	45 (85)
** **Secretor status	
** **Positive	88 (157)
** **Negative	12 (21)
** **Blood group	
** **O	62 (111)
** **A	20 (35)
** **B	16 (29)
** **AB	2 (3)

Data on breast-feeding duration were missing for 3 children, and data on secretor status and blood group were missing for 16 children.

Over the 3-year study period, 1454 stool samples were collected, of which 438 (30%) were collected during an episode of acute diarrhea, while 1016 (70%) samples were collected from children without diarrheal symptoms for at least 14 days. There was a median of 7 samples (range, 1–20 samples) collected per child. There were no major demographic differences in children with ≥7 samples collected, compared with those with <7 samples collected, with the exception of children with older mothers, who had fewer samples collected (*P* = .008).

### Diarrhea and Norovirus Infections

**Figure 1.**
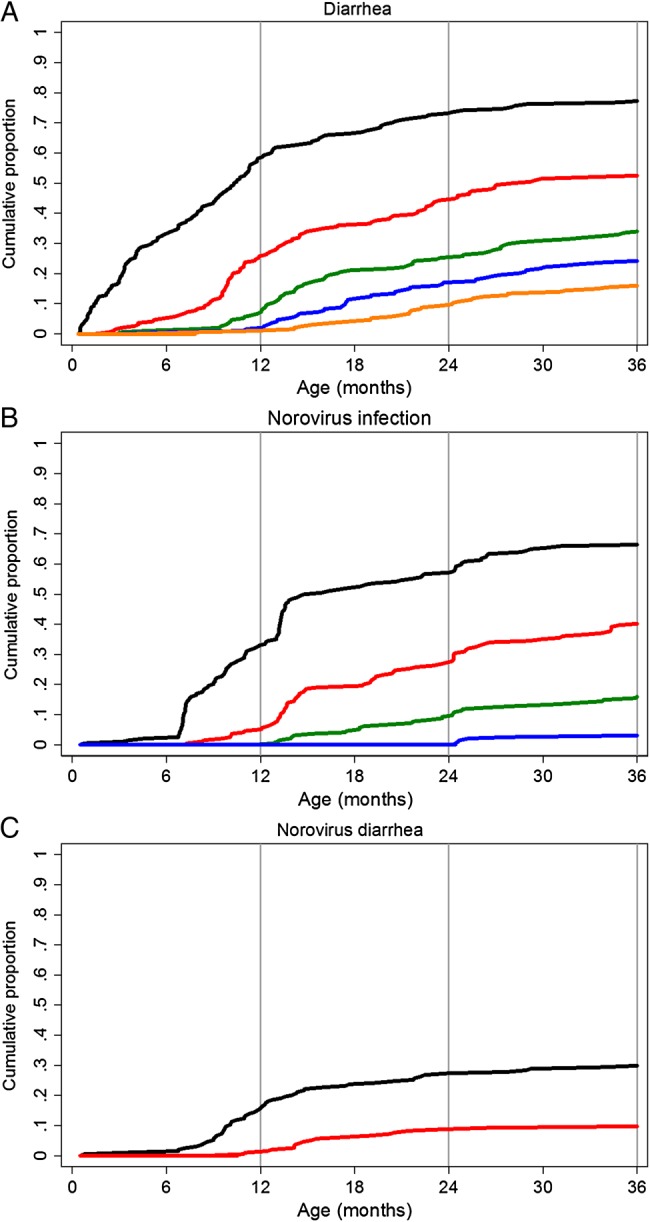
Cumulative incidence of diarrhea (*A*), norovirus infection (*B*), and norovirus diarrhea (*C*) from birth to age 36 months. First event, black; second event, red; third event, green; fourth event, blue; and fifth event, orange.

Over the 3-year period, we detected a mean (±SD) of 2.26 ± 2.30 diarrheal cases per child (range, 0–12 cases): 44 children (23%) had 0 episodes of diarrhea, 85 (44%) had 1–2 episodes, 50 (26%) had 3–5 episodes, and 15 (8%) had >5 episodes. The cumulative incidence of diarrhea in the surveillance cohort, by number of episodes over the 36 months of follow-up, is shown in Figure 1*A*.

**Figure 2.**
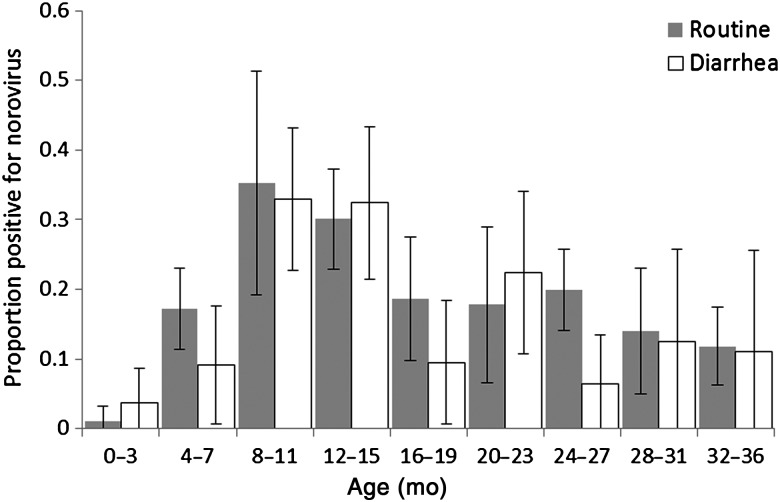
Norovirus detection among asymptomatic routine and diarrheal stool samples, by age.

Norovirus was detected in 260 samples (18%) but was not found more frequently in diarrheal samples (79 of 438 [18%]) than in routine samples (181 of 1016 [18%]; *P* = .919). This pattern did not appear to differ by age (Figure 2). However, when norovirus was detected, viral loads (based on Ct values) were higher in diarrheal samples (median Ct value, 28.02), compared with routine samples (median Ct value, 32.2; *P* = .005 by the Wilcoxon rank sum test; data not shown).

Sixty-six percent of children had at least 1 documented infection (with or without symptoms) with norovirus during the first 3 years of life, 40% of children had 2 infections, but few (16%) had >2 infections (Figure 1*B*). At least 1 episode of norovirus-associated diarrhea was observed in 30% of children (59), but relatively few (10%) had >1 episode (Figure 1*C*). Overall, norovirus infection and disease was detected at rates of 51 cases per 100 person-years (95% CI, 45–58) and 17 cases per 100 person-years (95% CI, 14–21), respectively.

Age was an important determinant of the risk of norovirus infections (Table 2): the peak incidence of norovirus infections, 76 infections per 100 person-years, was observed between 6 and 11 months of age, declining to 9.5 infections per 100 person-years at 24–36 months of age (Table 2). The associations between risk of norovirus infection or norovirus diarrhea and previous norovirus infections, sociodemographic factors, and individual characteristics are shown in Table 2. The only factor associated with the incidence of norovirus infections was season, with higher rates of infection during the dry months of July through November (*P* = .012). Previous norovirus infections were not significantly associated with the risk of subsequent norovirus infection.

**Table 2.** Association Between Previous Infections, Sociodemographic Characteristics, and Host Characteristics and Norovirus Infection and Disease Risk

Variable	Infection				Disease			
	No.	Rate^a^	RR (95% CI)^b^	*P* Value	No.	Rate^a^	RR (95% CI)^b^	*P* Value
No. of previous norovirus infections								
0	130	43.5	Reference		40	13.4	Reference	
1	79	71.5	1.01 (.51–1.99)	.972	30	27.1	1.88 (1.11–3.21)	.019
≥2	43	51.9	0.82 (.26–2.52)	.422	13	15.7	1.19 (.50–2.90)	.692
No. of previous genogroup I infections								
0	190	47.6	Reference		62	15.5	Reference	
≥1	62	66.6	1.21 (.90–1.63)	.204	21	22.6	1.51 (.84–2.74)	.170
No. of previous genogroup II infections								
0	161	47.7	Reference		52	15.4	Reference	
≥1	91	58.7	0.92 (.60–1.42)	.721	31	20.0	1.24 (.71–2.18)	.450
Age, mo								
0–5	5	5.2	Reference		3	3.1	Reference	
6–11	73	75.7	14.65 (5.92–36.24)	<.001	34	35.3	11.34 (3.49–37.01)	<.001
12–23	117	63.8	12.35 (5.05–30.23)	<.001	38	20.7	6.66 (2.06–21.59)	.002
24–36	57	49.1	9.50 (3.81–23.71)	<.001	8	6.9	2.16 (.57–8.17)	.243
Sex								
Male	106	46.1	Reference		28	12.2	Reference	
Female	146	55.5	1.20 (.93–1.55)	.153	55	20.9	1.68 (1.03–2.77)	.039
Socioeconomic status								
Low	32	39.1	Reference		10	12.2	Reference	
Medium	78	46.4	1.17 (.78–1.78)	.440	25	14.9	1.23 (.56–2.70)	.611
High	142	58.5	1.47 (1.00–2.17)	.049	48	19.8	1.60 (.77–3.33)	.208
No. of other children in household								
0	72	51.3	Reference		21	15.0	Reference	
1	58	45.5	0.89 (.63–1.27)	.537	19	14.9	1.04 (.51–1.95)	.902
≥2	122	54.3	1.02 (.76–1.38)	.885	43	19.1	1.21 (.72–2.77)	.505
Maternal education level								
Incomplete primary	19	36.2	Reference		6	11.4	Reference	
Complete primary	150	51.3	1.34 (.83–2.19)	.235	55	18.8	1.50 (.60–3.75)	.386
Complete secondary	83	56.1	1.52 (.91–2.52)	.106	22	14.9	1.24 (.47–3.31)	.655
Season								
Dry (July to Nov)	132	54.1	Reference		32	13.1	Reference	
Wet (Dec to Jun)	120	48.2	0.72 (.56–.93)	.012	51	20.5	1.36 (.87–2.16)	.179
Secretor status								
Negative	24	45.9	Reference		8	15.3	Reference	
Positive	216	52.8	1.15 (.75–1.77)	.522	71	17.4	1.20 (.53–2.71)	.655
Secretor genotype								
Not applicable^c^	24	45.9	Reference		8	15.3	Reference	
Heterozygous	104	50.4	1.09 (.70–1.71)	.703	27	13.1	0.89 (.37–2.09)	.793
Homozygous	112	55.2	1.21 (.77–1.90)	.402	44	21.7	1.51 (.66–3.47)	.328
Blood group								
O	149	52.1	Reference		47	16.4	Reference	
A	47	52.5	0.99 (.71–1.40)	.992	10	11.2	0.65 (.31–1.36)	.259
B	40	51.4	0.96 (.67–1.37)	.806	21	27.0	1.59 (.89–2.86)	.116
A/B	4	50.8	1.07 (.38–2.97)	.900	1	12.5	1.06 (.12–9.15)	.952

Abbreviations: CI, confidence interval; RR, relative risk.

^a^ Per 100 person-years.

^b^ All models include age and sex as controlling covariates.

^c^ Data are for secretor-negative children.

The incidence of norovirus diarrhea was strongly dependent on age, with a peak at 6–11 months of age of 35.3 episodes per 100 person-years. Norovirus diarrhea incidence was more frequent among females (*P* = .039) but was not significantly associated with secretor status or genotype. Having one previously documented norovirus infection was positively associated with the risk of norovirus diarrhea, although the same effect was not observed for ≥2 previous infections.

### Norovirus Genotype Distribution

A total of 154 samples (61%) were GII positive, 85 (34%) were GI positive, and 13 (5.2%) had GI/GII mixed infections. Factors associated with incidence of infection with GI and GII norovirus are shown in Tables 3 and 4. There was a tendency for higher risk among secretor-positive children for GII infection (although the difference was not statistically significant) but no apparent association with GI, after control for age and sex. Similarly, previous norovirus infections, regardless of genogroup, were not associated with the incidence of subsequent infection. The only factor associated with infection was the reduced incidence of GII infections observed during the wet season (December through June; *P* < .001). An association in the opposite direction was observed for GI, but this was nonsignificant.

**Table 3.** Association Between Previous Infections, Sociodemographic Characteristics, and Host Characteristics and Norovirus Genogroup I and II Infection Risk

Variable	Genogroup I				Genogroup II			
	No.	Rate^a^	RR (95% CI)^b^	*P* Value	No.	Rate^a^	RR (95% CI)^b^	*P* Value
No. of previous norovirus infection								
0	47	15.7	Reference		90	30.1	Reference	
1	36	31.7	1.54 (.95–2.47)	.074	49	44.3	1.15 (.80–1.65)	.432
≥2	16	19.3	1.01 (.45–2.24)	.973	28	33.7	0.92 (.58–1.46)	.730
No. of previous genogroup I infection								
0	75	18.8	Reference		124	31.0	Reference	
≥1	23	24.7	1.12 (.69–1.83)	.623	43	46.2	1.31 (.91–1.89)	.139
No. of previous genogroup II infection								
0	61	18.1	Reference		110	32.6	Reference	
≥1	37	23.8	1.07 (.70–1.67)	.731	57	37.8	0.81 (.39–1.69)	.584
Season								
Dry (July to Nov)	37	15.1	Reference		99	40.6	Reference	37
Wet (Dec to Jun)	61	24.5	1.38 (.86–1.98)	.204	68	27.3	0.55 (.40–.75)	<.001
Secretor status								
Negative	12	22.9	Reference		12	22.9	Reference	
Positive	82	20.1	0.87 (.46–1.65)	.658	147	35.9	1.56 (.86–2.82)	.135
Secretor genotype								
Not applicable^c^	12	22.9	Reference		12	22.9	Reference	
Heterozygous	41	19.8	0.86 (.45–1.65)	.658	69	33.5	1.45 (.79–2.68)	.232
Homozygous	41	20.2	0.88 (.46–1.69)	.702	78	38.5	1.68 (.92–3.09)	.094
Blood group								
O	60	21.0	Reference		95	33.2	Reference	
A	17	18.9	0.90 (.52–1.54)	.692	32	35.7	1.07 (.71–1.59)	.753
B	15	11.6	0.90 (.51–1.59)	.826	29	37.2	1.10 (.72–1.69)	.658
A/B	2	6.2	1.27 (.30–5.37)	.742	3	37.6	1.22 (.38–3.90)	.737

Abbreviations: CI, confidence interval; RR, relative risk.

^a^ Per 100 person-years.

^b^ All models include age and sex as controlling covariates.

^c^ Data are for secretor-negative children.

**Table 4.** Norovirus Genotype–Specific Rates of Infection for Secretor-Negative and Secretor-Positive Children

Variable	Secretor Negative		Secretor Positive		RR (95% CI)^b^	*P* Values
	Infections, No.	Rate^a^	Infections, No.	Rate^a^		
GI	4	7.6	18	4.4	0.61 (.21–1.70)	.342
GII (excluding GII.4)	7	13.4	38	9.0	0.69 (.31–1.15)	.358
GII.1	5	9.6	17	4.2	0.43 (.15–1.21)	.111
GII (untypeable)	0	0	6	1.5	Undetermined	<.001
GI/GII mixed (excluding GII.4)	3	5.7	4	1.0	0.19 (.05–.80)	.024
Non-GII.4 (combined)	14	26.8	59	14.4	0.56 (.34–.94)	.029
GII.4 (single infections)	0	0	27	6.6	Undetermined	<.001
GII.4 (including GI mixed)	0	0	31	7.6	Undetermined	<.001
GI/GII mixed (including GII.4)	0	0	4	1.0	Undetermined	<.001

Abbreviations: CI, confidence interval; GI, genogroup I; GII, genogroup II; GII.1, genogroup II, genotype 1; GII.4, genogroup 2, genotype 4; RR, relative risk.

^a^ Per 100 person-years.

^b^ All models include age and sex as controlling covariates.

**Figure 3.**
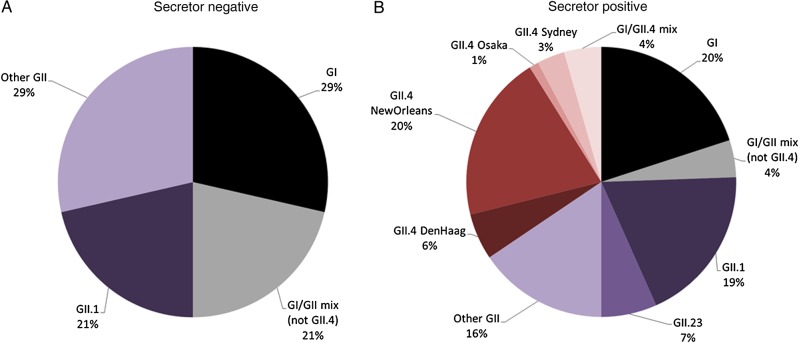
Norovirus genotype distribution of infections in secretor-negative (*A*) and secretor-positive (*B*) children. Abbreviation: G, genogroup.

We were able to determine the genotype for 106 samples (Figure 3). The most common genotype was GII.4 (27 infections [25%]), followed by GII.1 (20 [19%]), GI.3 (9 [8%]), GII.2 (11 [10%]), and a tentative new GII genotype (GII.23; 6 [6%]). We observed 12 GI/GII mixed infections (11%), including 4 due to GII.4 (4%). Among the GII.4-positive samples (including those from mixed infections), the following variants were observed: New Orleans 2009, 21 samples; Den Haag 2006, 5; Sydney 2012, 4; and Osaka 2008, 1. The genotype distribution among diarrheal stools and healthy stools was similar.

All 27 GII.4 single infections and all 4 coinfections that were positive for GII.4 were in secretor-positive children (relative risk [RR], undetermined; *P* < .001 for both comparisons; Tables 3 and 4). We observed a nonsignificantly lower rate of GII infection (excluding GII.4; RR, 0.69; 95% CI, .3–1.2) and GI infection (RR, 0.61; 95% CI, .2–1.7) among secretor-positive children. However, when combining all non-GII.4 noroviruses, the rates of infection were significantly lower among secretor-positive children, compared with secretor-negative children (RR, 0.56; 95% CI, .3–.9; *P* = .023). We also detected a tentative new GII genotype (GII.23); all 6 positive samples were detected in secretor-positive children (RR, undermined; *P* < .001).

## DISCUSSION

With 60% of children having at least 1 infection (symptomatic or asymptomatic) and 30% at least 1 norovirus-associated diarrhea episode within the first 3 years of life, this study confirms the high incidence of norovirus infection in children that has been found by many studies worldwide [1, 4, 30]. Our results were not consistent with the notion of acquired immunity to norovirus, whereby each infection would be expected to provide a degree of protection against the next. Nor did we find evidence that immunity is genogroup specific: rates of GI or GII infections did not decrease following previous infection(s) with the same genogroup. Overall, the rates of norovirus infection and disease were similar among secretor-positive and secretor-negative children, but the genotypes causing infections differed markedly. All GII.4 infections were among secretor-positive individuals.

Our findings contrast, to some extent, with those of a recent birth cohort study from neighboring Peru [4]. Saito et al found roughly similar rates of norovirus infection and disease that we found, but they observed a modest reduction in infection and disease rates in children with previous infections. Saito et al tested routine samples more frequently, at least in the first year of life, when monthly samples were tested, which means they had a more complete capture of asymptomatic infections. Field workers also visited each household twice weekly, so they may have had more-complete capture of diarrhea episodes.

Our study is consistent with volunteer challenge studies and observational studies demonstrating that secretor-negative individuals have near-complete resistance to GII.4 infection [15, 31], as well as to GI.I. infection [8, 10] and a new GII genotype described in this study. GII.4 noroviruses can also infect secretor-negative individuals, suggesting a lack of complete resistance to disease [16]. This is in agreement with a recent study that suggests that most recent GII.4 variants are able to bind Lewis glycans from secretor-negative individuals [14]. Challenge studies with norovirus GII.2 showed that 33% of secretor-negative individuals were infected [17]. Moreover, other studies have shown that GI.3 noroviruses can infect independently of secretor status of the host [18, 19]. Resistance to infection among secretor-negative individuals has been demonstrated mainly for GI.1 and GII.4 [8, 10, 15], with an apparent lack of association for GII.3 [31] or GII.2 [11]; few data exist for other genotypes.

An unexpected finding of our study was that the rate of non-GII.4 infection was nearly double among secretor-negative children. Therefore, overall, rates of disease were the nearly same among secretor-positive and secretor-negative children. We are not aware of any previous data suggesting a heightened risk of infection for secretor-negative individuals. It is possible that non-GII.4 viruses bind to currently unknown ligands in secretor-negative individuals. Another hypothesis is that GII.4 infection confers some degree of heterotypic immunity to subsequent infection with non-GII.4 viruses. However, our sample size was too small to test this hypothesis.

Birth cohort studies can be instrumental in gaining understanding of the acquisition of immunity to childhood infections [32, 33], and we have advocated for such study designs for norovirus [34]. So why did we not observe patterns consistent with the acquisition of immunity? A weakness of our study was the relatively infrequent collection of routine stool samples from healthy children. We collected a routine stool sample approximately every 6 months. With duration of shedding typically lasting <1 month, more-frequent sampling would result in detection of more infections, so it is likely that substantial numbers of asymptomatic infections were missed. Although there were extended periods when some children were temporarily lost to follow-up, excluding these periods from analyses did not appreciably change our results (data not shown), but this leaves open the possibility that substantial numbers of symptomatic infections were missed. Underascertainment in our cohort is a possible explanation for our negative findings: we measured a diarrhea incidence of <1 episode per child-year, compared with an estimate of 4.0 episodes per child-year based on a systematic review of 29 studies from the Americas [35].

In summary, our study suggests a high incidence of norovirus infection and disease in Ecuadorian children, but we did not find clear evidence for acquired immunity. Secretor-negative individuals were completely protected against GII.4 viruses, as well as a tentative new GII genotype, and they had higher rates of infection with non-GII viruses. Taken together, these findings point to a more complicated relationship between host genetics and pathogen than previous described, with GII.4 being more common and other viruses being less common among secretor-positive individuals.

Future studies may require more-frequent sampling of asymptomatic infections, longer follow-up, and larger sample sizes to improve our understanding of the acquisition of immunity and empirical estimates of the duration of protection, if such protection truly exists. Because our understanding of norovirus immunity comes mainly from controlled clinical trials rather than from observational studies in the field, it is possible that natural infection may differ in a number of ways, including exposure frequency, patient age, exposure dose, and norovirus genotype. Much larger studies will be required to explore norovirus genotype–specific immunity. As such, further observational field studies are necessary to understand both naturally acquired and vaccine-acquired immunity.
